# Facial Aesthetic Laser-Assisted Protocol for the Management of Acne and Pigmentation: A Case Report

**DOI:** 10.7759/cureus.28871

**Published:** 2022-09-06

**Authors:** Nancy Zeaiter, Kinga Grzech-Leśniak, Zuzanna Grzech-Leśniak, Maher Ghandour, Marwan El Mobadder

**Affiliations:** 1 Department of Plastic and Reconstructive Surgery, Lebanese University, Beirut, LBN; 2 Department of Dental Surgery, Wroclaw Medical University, Wroclaw, POL; 3 Department of Periodontics, School of Dentistry, Virginia Commonwealth University, Virginia, USA; 4 Department of Orthopedics and Traumatology, Heidelberg University Hospital, Heidelberg, DEU

**Keywords:** aesthetic medicine, laser treatment, laser in dermatology, dental laser therapy, aesthetic facial surgery

## Abstract

The demand for aesthetic procedures is significantly increasing worldwide. In this case report, an in-office laser-assisted protocol coupled with rejuvenating concentrate serum (Gluage, TEBISKIN Gluage, SkinMed, Italy) was made. A 24-year-old female patient presented with a chief complaint of abundant facial acne and localized pigmentation. Clinical examination revealed the presence of abundant acne on the forehead and cheeks and the presence of localized pigmentation. Laser-assisted protocol coupled with rejuvenating concentrate serum was suggested. The protocol consisted of a thorough cleansing of the face followed by irradiation with a 980 nm diode laser (Smart M, Lasotronix, Poland), followed by a 405 nm diode laser (Smart M, Lasotronix, Poland), the application of rejuvenating concentrate serum (Gluage, TEBISKIN Gluage, SkinMed, Italy), and irradiation with the 635 nm diode laser (Smart M, Lasotronix, Poland). The protocol was made once per week for three weeks (three sessions in total), and a three-month follow-up was made after the end of the last session to confirm the effectiveness of the treatment. Stomatology 1 diode laser (Smart M, Lasotronix, Poland) was used in this case report as a 980 nm, 405 nm, and 635 nm diode laser (Smart M, Lasotronix, Poland). During the follow-up period, an almost total reduction of the acne was observed with the total disappearance of the localized pigmentation. This case report confirms the effectiveness of the proposed laser-assisted facial aesthetic treatment. We invite further studies to be made within the same suggested promising protocol.

## Introduction

In recent decades, the demand for aesthetic procedures has significantly increased worldwide. Facial aesthetic procedures remain one of the most demanded and desired procedures due to their direct impact on the overall perception of beauty [1,2]. Among available treatments in facial aesthetics, minimally invasive techniques are being more and more popular due to their relatively low risk of complications and their quicker recovery time compared to surgical interventions [2-4]. In this context, lasers with specific wavelengths and parameters and if applied within a certain protocol can show imposing results on the overall facial treatment [5]. For instance, a plethora of studies demonstrated that the use of lasers can stimulate the formation of collagen fibers and fibroblasts, increases local vascularization, and significantly disinfect tissues due to their bactericidal effect [5-9]. In addition, if used adequately, lasers can increase the absorption of serums, leading to a better reaction and hence better results [10]. Again, to obtain these results, conditions such as the use of a proper laser wavelength, optimal parameters, and protocols must be applied [11]. The aim of this case report was to assess an innovative minimally invasive approach for the treatment of facial acne and pigmentation. The treatment protocol consisted of an in-office laser-assisted facial treatment with three different wavelengths, 980 nm, 405 nm, and 635 nm (Smart M, Lasotronix, Poland), coupled with rejuvenating concentrate serum application.

## Case presentation

**Figure 1:**
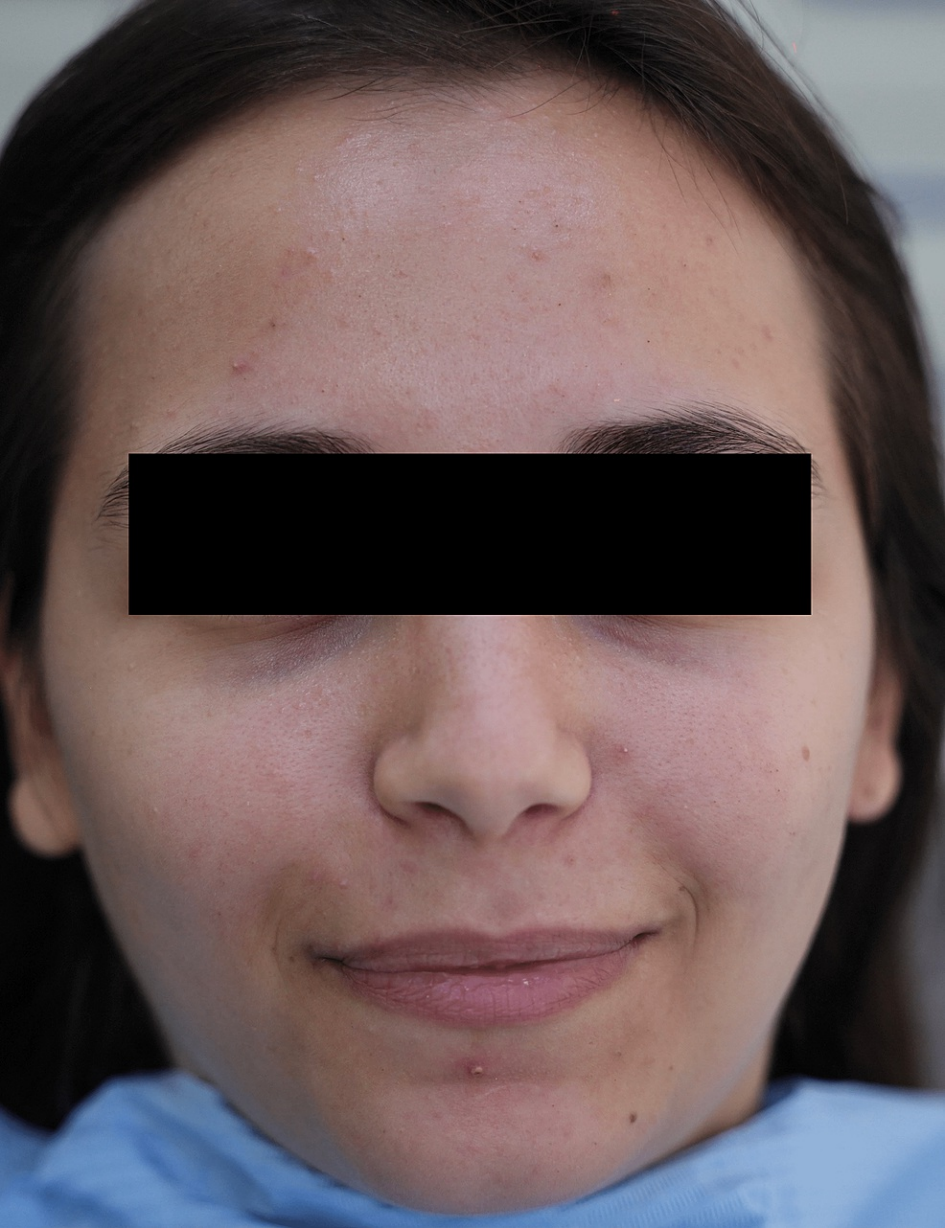
Clinical aspect of the face before any intervention.

A 24-year-old female with no systemic condition presented to our polyclinic complaining of her unaesthetic facial appearance. Clinical diagnosis revealed the presence of excessive acne on the forehead and both cheeks and a few localized pigmentation (Figure 1). As a therapeutic option, a laser-assisted facial aesthetic protocol based on the use of three different wavelengths coupled with rejuvenating concentrate serum was suggested. The patient confirmed the acceptance of the treatment, and written informed consent was obtained from her after explaining the steps, possible side effects, and limitations of the procedure. Moreover, written informed consent was obtained from the patient stating that all facial photos of this case can be published.

Treatment protocol

The protocol used in this clinical study was based on the application of three different laser wavelengths in adjunction to the use of a rejuvenating concentrate serum with acetylglucosamine, taurine, ectoine, and hyaluronic acid as active ingredients (TEBISKIN Gluage, SkinMed, Italy). Specific protective eyeglasses for the patient, assistant, and operator were worn in each step according to the wavelength used. To simplify the treatment, the forehead was considered the first part of the face; the right cheek, right part of the chin, and right part of the nose were considered the second part of the face; and the left cheek, left part of the chin, and left part of the nose were considered the third part of the face. In this way, the face was virtually divided into three parts.

Skin Cleansing

Thorough skin cleansing was made as a preparatory stage before laser irradiation. The aim was to remove impurities from the skin and promote the penetration of light and cream into the soft tissues. Skin cleansing gel (Tebiskin Sooth-Clean, Tebiskin, SkinMed, Italy) was spread on the face, left for about 30 seconds, and then rinsed thoroughly with water.

Irradiation With 980 nm Diode Laser

A 980 nm diode laser (Smart M, Lasotronix, Poland) was used after skin cleansing. The parameters of irradiation were as follows: power of 8 W, continuous mode, noncontact mode with a distance of 1 cm from the target, and a tip of 200 µm core diameter and 200 cm length. Irradiation was made for 540 seconds (nine minutes) on the total surface of the face. The period of time was divided into 180 seconds for each part of the face (the face was divided into three parts as explained above). The aim of the irradiation with the 980 nm diode laser was to warm up the face to allow the cream to penetrate deeper layers of the skin.

Irradiation With 405 nm Diode Laser

After the use of the 980 nm wavelength, a 405 nm diode laser was used (Smart M, Lasotronix, Poland). The exact same protocol as described with the 980 nm diode laser was used for the 405 nm wavelength but with the following parameters: power of 320 mW/cm^2^, energy density of 30 J/cm^2^, continuous mode, and a contact mode with a 14 mm applicator tip, for three minutes per quadrant. The aim of the 405 nm wavelength was to increase the disinfection of the skin by killing bacteria, viruses, and fungi.

Application of the Gluage Serum

**Figure 2:**
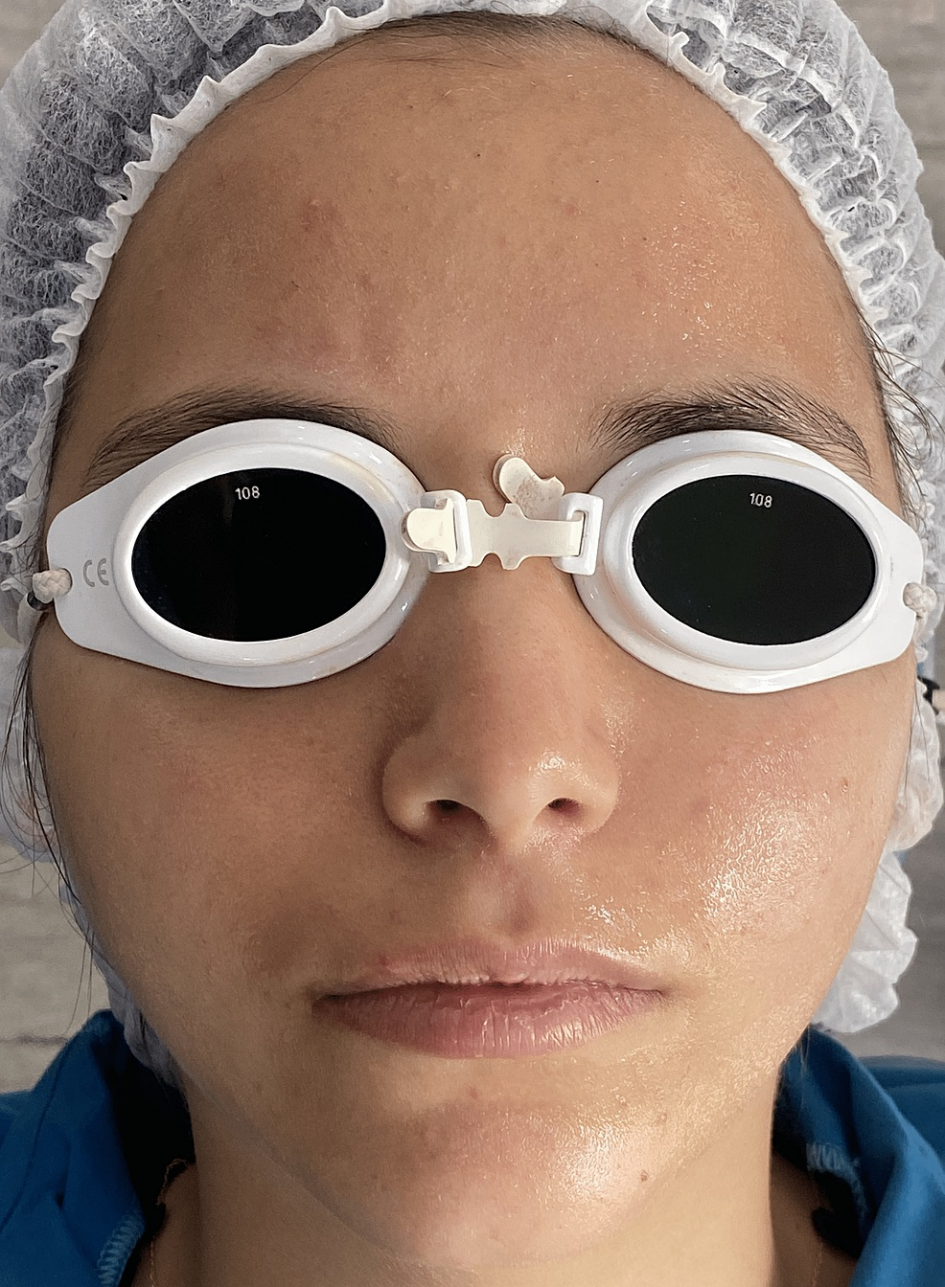
Clinical aspect of the face after Gluage serum application.

At this stage of the treatment, Gluage serum was applied. The ampoule of the bottle (Cytoceuticals, TEBISKIN Gluage, SkinMed, Italy) was mixed properly with vitamin C for about one minute before applying it to the face. The gloves of the operator were removed, and then, the serum was applied to the previously irradiated area of the face, and in a gentle massage, the serum was spread on the totality of the face in a way to obtain a thin layer visible to the naked eye (Figure 2).

Irradiation With 635 nm Diode Laser

After a wait of three minutes, the 635 nm wavelength was used (Smart M, Lasotronix, Poland). The protocol was exactly the same as with the 980 nm and 405 nm but with different parameters. The parameters at this point were as follows: power of 400 mW, energy dose of 20 J/cm^2^, continuous mode, contact mode, and a tip of 14 mm. The aim was to stimulate the production of collagen fibers and improve the penetration of the active serum ingredients, therefore moistening further the skin. At this point, a post-laser care face cream (Tebiskin Post Laser Care (PLC), SkinMed, Italy) was applied, and the face was massaged thoroughly for three minutes.

Results of the treatment

**Figure 3:**
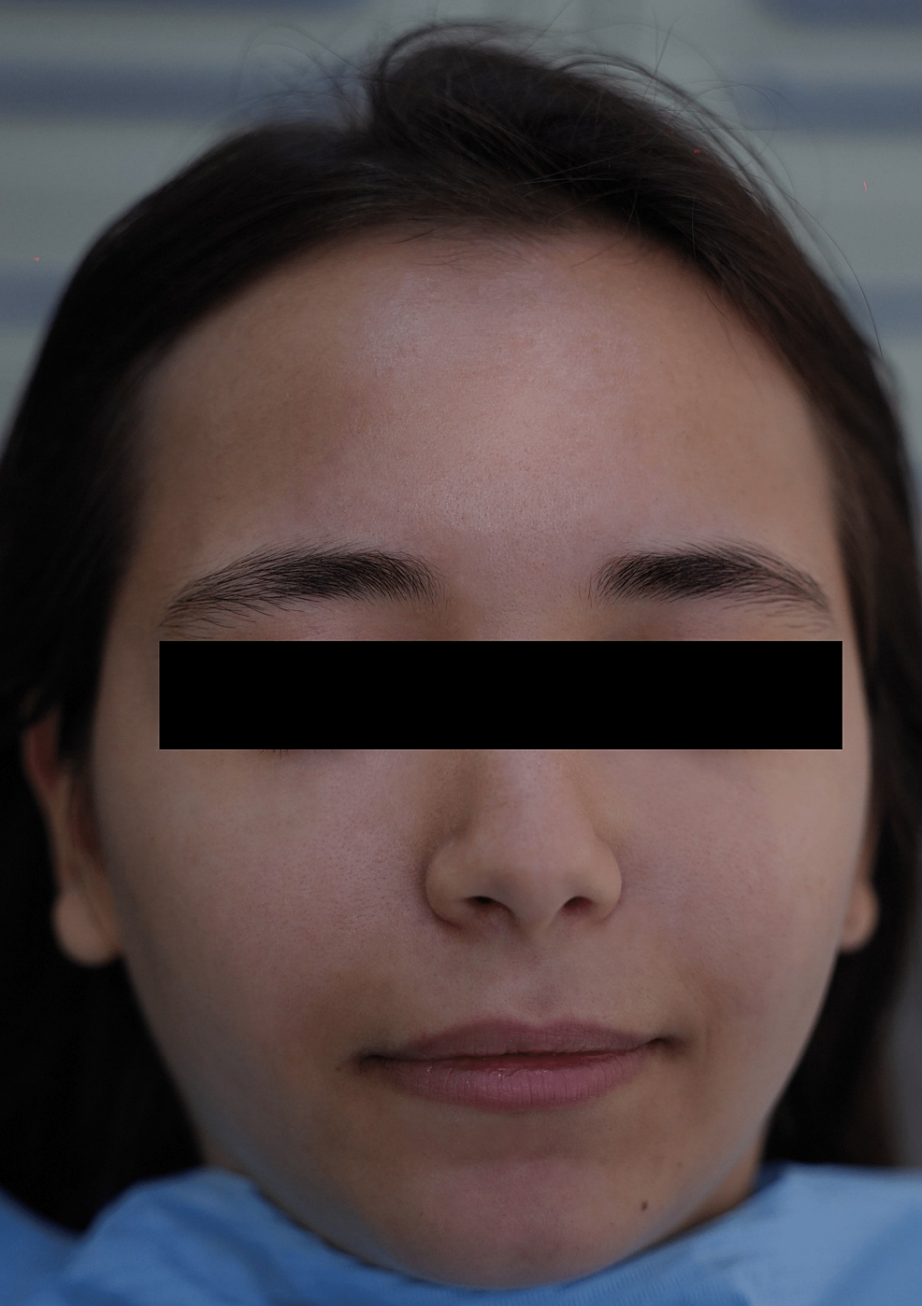
Clinical aspect of the face 48 hours after the third session.

After a total of three sessions done in a period of three weeks (one session per week), there was almost a total disappearance of the acne and facial pigmentation (Figure 3). The patient and the operator were satisfied with the results obtained after each application, and finally, at the third session, a decision was made to end the treatment because of the satisfactory results obtained. Moreover, this protocol did not show any side effects, pain, or discomfort during and/or after treatment and within three months of follow-up.

## Discussion

This case report showed that the suggested treatment protocol combining three different wavelengths and rejuvenating serum application results in a significant improvement in facial aesthetics. As a result, there was almost a total disappearance of the acne and pigmentation. Furthermore, both the patient and the operator were satisfied with the treatment, and no side effects or discomfort were seen. Hence, the treatment can be considered significantly effective and safe.

Several facts can explain the findings obtained in our study. The 980 nm diode laser used at the beginning of the treatment served as skin preparation and warm-up, which must have increased later the absorption of the serum [12,13]. The heating done by the 980 nm diode laser must have led to pore opening [12,13]. Hence, the cream was able to penetrate deeper layers of the skin. The 405 nm wavelength was used with the aim of increasing disinfection [14,15]. In fact, the 405 nm wavelength cleanses the skin by its well-documented bactericidal effect and its strong action against bacteria, viruses, and fungi [16]. Since acne-prone skin is known to have more bacterial load, disinfection with the 405 nm wavelength must have been a key in treating the acne. In addition, the 635 nm wavelength of the diode laser was used after applying the Gluage gel as a photobiomodulation (PBM) therapy. PBM is the therapeutic use of light to stimulate a nonthermal modulation of the living tissue [17]. This stimulated the collagen production in the skin and improved the penetration of serum substances. This stimulation effect is obtained with the 635 nm wavelength [17]. It is now well demonstrated that PBM’s mechanism of action is based on the stimulation of the cytochrome C oxidase enzyme, resulting in an increase in ATP production [17,18]. In this protocol, PBM therapy with 635 nm wavelength led to a stimulation of collagen fibers and vasodilatation and therefore resulted in a stimulation of the healing process and the attenuation of the inflammatory process. Accordingly, this case report confirms that if the laser was used according to the suggested protocol, a significant improvement in facial aesthetics can be seen. Several studies assessing the effectiveness of the diode laser in the management of facial acne and pigmentation can be found in the literature. For instance, Rathod et al. showed that 1,450 nm diode laser is effective and well endowed in facial acne scars when utilized with double-pass at low energy [19]. However, in contrast to our study, five sessions were used, while in our protocol, only three sessions were needed for a satisfactory result. Moreover, other wavelengths can be used in the treatment of facial acne and pigmentation, such as the carbon dioxide (CO_2_) laser. In this context, Manuskiatti et al. showed that the use of carbon dioxide laser can notably improve atrophic acne scars [20]. In their systematic review, they showed that combination remedies should be appraised for ice pick-type acne scars, and the use of dermocosmetics in preoperative and postoperative care may be helpful to patients [20]. This is in accordance with our study, in which three different wavelengths and rejuvenation serum application were used to obtain a satisfactory result. However, one of the limitations of our study is that this protocol was made on only one patient. Therefore, further investigations are invited to be done within our treatment protocol and parameters.

## Conclusions

The demand for aesthetic procedures is significantly increasing worldwide. Minimally invasive techniques used in enhancing facial aesthetics are becoming more popular due to their lower complication rates and faster onset of recovery compared to surgical interventions. According to this case report, the protocol consisting of an in-office laser-assisted facial treatment with three different wavelengths, 980 nm, 405 nm, and 635 nm (Smart M, Lasotronix, Poland), coupled with rejuvenating concentrate serum application, can be considered a safe and effective treatment for facial acne and pigmentation.
